# Facile fabrication of quaternized chitosan-incorporated biomolecular patches for non-compressive haemostasis and wound healing

**DOI:** 10.1016/j.fmre.2023.05.009

**Published:** 2023-05-30

**Authors:** Zesheng Chen, Yixuan Zhang, Kexin Feng, Tao Hu, Bohan Huang, Jinlan Tang, Junjie Ai, Liang Guo, Weikang Hu, Zijian Wang

**Affiliations:** a Department of Urology, Cancer Precision Diagnosis and Treatment and Translational Medicine Hubei Engineering Research Center, Zhongnan Hospital of Wuhan University, Wuhan 430071, China; b Ministry of Education Key Laboratory of the Green Preparation and Application for Functional Materials, Hubei Key Laboratory of Polymer Materials, School of Materials Science and Engineering, Hubei University, Wuhan 430062, China; c Department of Plastic Surgery, Zhongnan Hospital of Wuhan University, Wuhan 430071, China; d Department of Clinical Laboratory, Hubei Provincial Hospital of Traditional Chinese Medicine, Wuhan 430061, China

**Keywords:** Quaternized chitosan, Patches, Biocompatible, Antibacterial, Haemostatic, Wound healing

## Abstract

Cell-free wound dressings (WDs) with desirable effectiveness and safety have received much attention in the field of regenerative medicine. However, the weak linkages between bioactive polymers and the spatial structure of WDs frequently result in interventional treatment failure. Herein, we create a series of quaternized chitosan (QCS)-incorporated composite hydrogels (referred to as GHCH-n) by UV cross-linking and then convert them into microneedle patches (MNPs). QCS, which is positively charged and amphiphilic, is essential for broad-spectrum antibacterial and haemostatic activities. QCS is proven to be slightly toxic, so it is immobilized into the methacrylate gelatine (GelMA) molecular cage to minimize adverse effects. A polydimethylsiloxane micro-mould is used to shape the MNPs. MNPs can pierce tissue, seal off bleeding sites, and cling to wounds securely. Thus, MNPs can cooperate with GHCH-n hydrogels to halt bleeding and accelerate wound healing. This study recommends GHCH-10 MNPs as an advanced biomaterial. Several preclinical research models have thoroughly validated the application effect of GHCH-10 MNPs. This research also proposes a novel strategy for integrating the nature of bioactive polymers and the structure of composite biomaterials. This strategy is not only applicable to the fabrication of next-generation WDs but also shows great potential in expanding interdisciplinary domains.

## Graphical abstract

**Figure:**
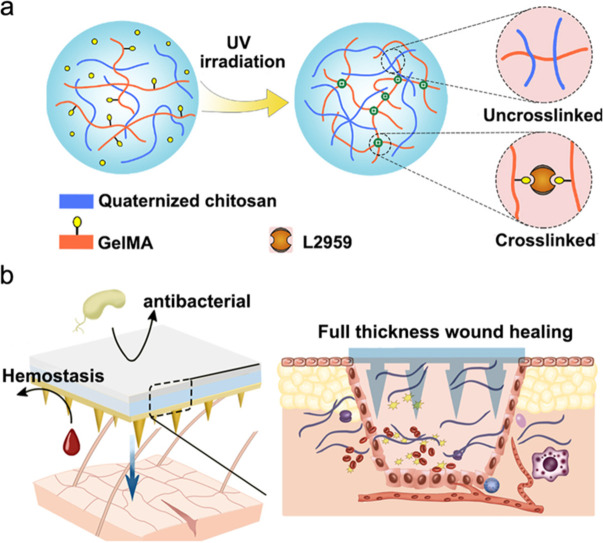

## 1 Introduction

The skin is the human body's initial line of defence against external threats [1,2]. When the integrity of the skin is destroyed, the wound-healing process immediately starts and usually lasts for days to months [3]. Wound healing is a dynamic and overlapping process that is divided into four phases: haemostasis, inflammation, proliferation, and remodelling [4,5]. It can be easily disturbed by many negative factors, such as secondary injury, bacterial infection, hyperglycaemia, and hypoxia [6,7]. Thus, the clinical needs of wound healing are quite complicated depending on the types of wounds and intervention factors. In recent decades, the fields of biomedical engineering have made great progress in combating skin injury [8], [9], [10]. However, multifunctional biomaterials that can play a distinctive and differentiated role in different phases of wound healing are still lacking.

Hydrogels feature a 3D network structure similar to that of the extracellular matrix (ECM) and are thus regarded as one of the most valuable wound dressings (WDs) [8,^11^,12]. Based on clinical needs, ideal hydrogel-based WDs should have good biocompatibility and haemostatic ability, broad-spectrum antibacterial, and pro-regenerative activities [13,14]. It is extremely difficult to integrate all of the above functions into one platform of a biomolecular hydrogel. Chemical composition and spatial structure are both key factors determining the functions of biomaterials [15]. Researchers have developed a series of bioactive chemicals, such as silk fibroin (SF), polyglutamic acid (PGA), polylysine (PL), and chitosan derivatives (CSD), to fabricate next-generation biomaterials [16], [17], [18], [19], [20]. However, the spatial structure of biomaterials has not attracted enough attention. It is worthwhile to explore the synergistic relationships between the chemical composition and spatial structure of biomaterials [21,22].

Quaternized chitosan (QCS) is a bioactive derivative of natural chitosan [2,23]. Compared to natural chitosan, QCS has been modified with hydrophilic groups by direct quaternary ammonium substitution, epoxy derivative open loop, or N-alkylation [24]. QCS is positively charged, which can shield the opposite charge of red blood cells to promote coagulation [25]. Meanwhile, QCS is an amphiphilic long-chain molecule that can be inserted into the cell membrane to cause bacterial lysis and death [26]. As a potential haemostatic and antibacterial polymer, QCS has been preliminarily applied to hydrogel-based WDs [27,28]. However, QCS is water-soluble and chemically inert, making cross-linking difficult. Methacylated gelatine (GelMA) is a photoactive polymer and is widely used as the carrier of other biomolecules [29]. The GelMA and QCS composite hydrogel (named GHCH-n) can be facilely fabricated by sequential blending and UV curing.

The spatial structure of the GHCH-n hydrogel is further optimized to improve its application effect. Microneedle patch (MNP) is a novel physical penetration platform consisting of multiple micron-sized needle tips connected to a base in an array manner [30,31]. The unique structure of MNPs is conducive for transdermal drug delivery and tissue adhesion [32]. Compared to traditional syringe needles, MNPs do not reach deeper tissues. Thus, minimally invasive MNPs not only achieve high drug delivery efficiency but also minimize the risk of infection, bleeding, and pain [33]. As one of the frontiers of biomedical engineering, research on MNP-based haemostatic biomaterials has been increasingly reported. For example, Lee. et al. developed a tranexamic acid-loaded MNP for first-aid haemostasis [34]. The theory and method of MNP have been largely improved to reduce costs and increase repeatability, indicating a broad prospect of clinical transformation [35,36]. In this study, GHCH-n hydrogels were processed into MNPs by a micro-moulding technique. It is hypothesized that the GHCH-n MNPs could puncture into the tissue and seal off the bleeding sites safely. Meanwhile, the release of QCS from the GHCH-n MNPs can be sustained allowing it to play a vital role in combating bleeding, bacterial infection and wound healing. Notably, the haemostatic activity of GHCH-n MNPs could be simultaneously attributed to physical plugging and electrostatic shielding [37,38].

**Fig. 1:**
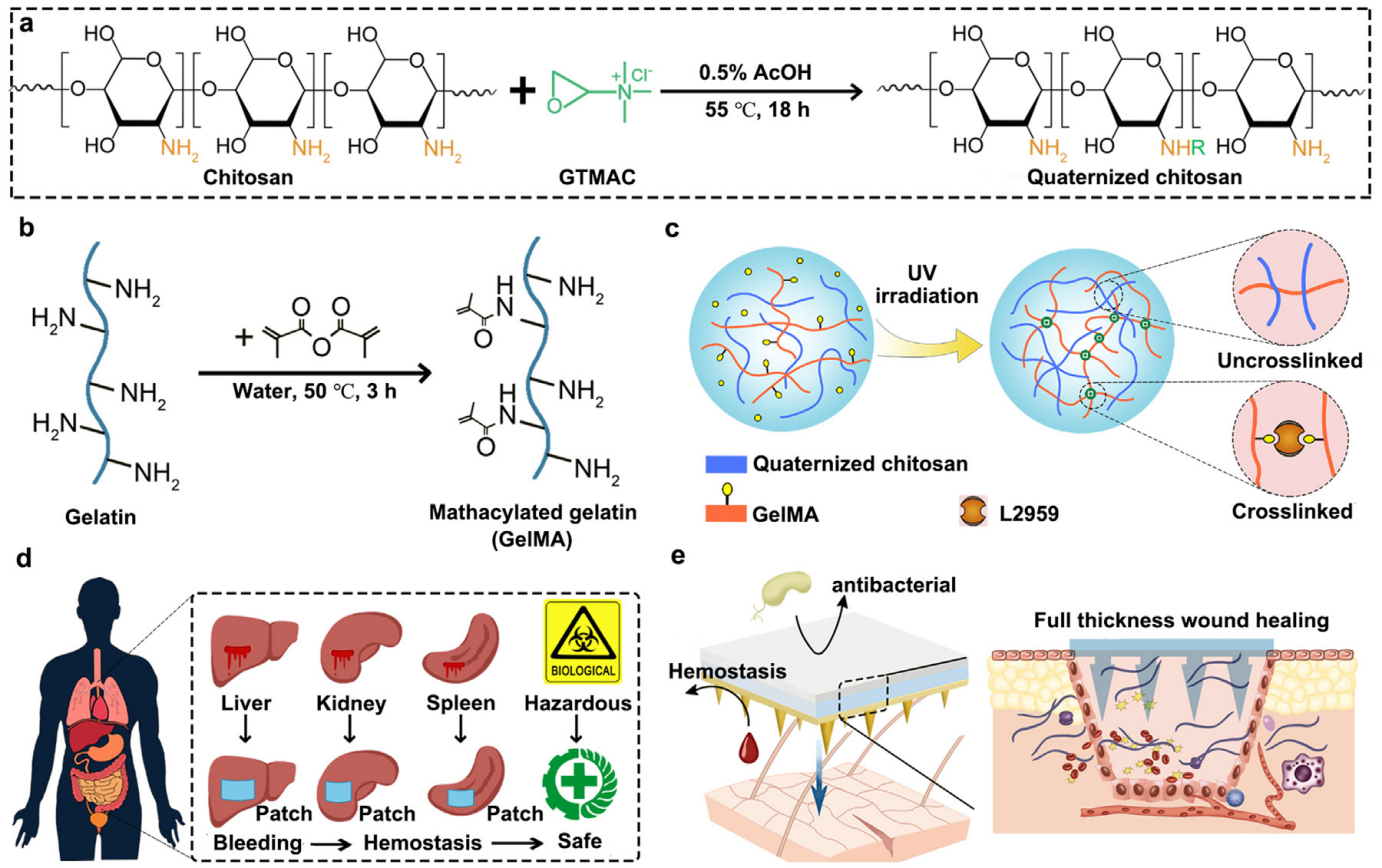
**Schematic illustration of quaternized chitosan-incorporated biomolecular patches and their biomedical applications.** (a) Chemical synthesis method of quaternized chitosan (QCS); (b) Chemical synthesis method of methacrylate gelatine (GelMA); (c) Ultraviolet (UV) cross-linked GelMA/QCS composite hydrogels (named GHCH-n) were prepared; (d) GHCH-n was haemostatic and biocompatible; (e) GHCH-n was shaped as microneedle patches for excellent full-thickness wound healing.

This research presents a novel strategy for integrating the nature of bioactive polymers and the structure of composite biomaterials, resulting in multifunctional biomolecular GHCH-n MNPs for complex wound healing. Fig. 1a-c depict the overall concept of this work. QCS and GelMA were chemically synthesized before being combined to create GHCH-n hydrogels and MNPs. As the foundation of translational research, this study also constructed a variety of preclinical models *in vivo* and *in vitro,* and the results confirm the products' efficacy and safety across several dimensions. The biocompatible, haemostatic, broad-spectrum antibacterial, and pro-regenerative properties of GHCH-n MNPs are highlighted in Fig. 1d-e. The products in this study are competitive when compared to other hydrogel-based WDs on the market.

## 2 Materials and methods

### 2.1 Materials

Gelatine and methacrylic anhydride (MA) were purchased from Macklin Chemical Reagent Co., Ltd. (Shanghai, China). Chitosan (CS) and glycidyl trimethylammonium chloride (GTMAC) were obtained from Sigma‒Aldrich Biochemical Co., Ltd. (Shanghai, China). The photo-initiator 2‑hydroxy-4′-(2-hydroxyethoxy)−2-methylpropiophenone (L2959), paraformaldehyde, paraffin, isoflurane, agarose, peptone, yeast extract, and NaCl were purchased from Sinopharm Co., Ltd. (Shanghai, China). Mouse lung fibroblasts (L929), human umbilical vein endothelial cells (HUVECs), and rat bone marrow mesenchymal stem cells (BMSCs) were kindly provided by the Medical Research Institute (MRI), Zhongnan Hospital of Wuhan University. *Escherichia coli* (*E. coli*) and *Staphylococcus aureus* (*S. aureus*) were purchased from China General Microbiological Culture Collection centre (Beijing, China). Dulbecco's modified Eagle's medium (DMEM), Roswell Park Memorial Institute-1640 (RPMI-1640) medium, foetal bovine serum, normal saline, trypsin solution, and phosphate-buffered saline (PBS) were purchased from Thermo-Fisher Scientific Co., Ltd. (Waltham, USA). A polydimethylsiloxane (PDMS) mould was customized by Zhongding Yuxuan New Material Technology Co., Ltd. (Anhui, China). A live/dead cell staining kit was purchased from Dojindo Co., Ltd. (Shanghai, China). Other chemicals were used without further purification.

### 2.2 Preparation of GelMA/QCS composite hydrogel

The GelMA was synthesized and characterized according to previous literature [39], and QCS was also synthesized and characterized following published literature [40] (see the Supplementary Material). Then, the obtained GelMA and QCS were dissolved in deionized water to prepare 20% GelMA and 2% QCS solutions. These solutions were blended at a specific proportion. The photo-initiator L2959 was added to the mixed solution to reach a final concentration of 0.05%. After centrifuging at 3000 rpm to remove air bubbles, the obtained solution was poured into a mould and cross-linked by UV irradiation at a power of 500 W for 180 s. Thus, the prepared GelMA and QCS composite hydrogels were coded as GHCH-n (*n* = 0, 5, 10, and 15, corresponding to the weight percentage of QCS). The codes and composites of GHCH-n are shown in Table 1.

**Table 1:** **The codes and compositions of the GelMA/QCS composite hydrogels**.

	20% GelMA (g)	2% QCS (g)	5% L2959 (g)	UV power (W)	UV time (s)
GHCH-0	100	0	1	500	180
GHCH-5	65.5	34.5	1	500	180
GHCH-10	47.3	52.7	1	500	180
GHCH-15	34.8	65.2	1	500	180

### 2.3 Fabrication of GHCH-10 MNPs

Microneedle patches (MNPs) were fabricated using a polydimethylsiloxane (PDMS) mould with a centre-to-centre interval of 700 µm and sharp needles tapering to a 10 µm radius and an 800 µm height. These needles were arranged in a 14 × 14 array at the base. The GHCH-10 as-gelled solution was added to the PDMS mould, and a pumping vacuum was applied for at least 5 min. After that, the samples were irradiated with UV at a power of 500 W for 180 s. The resultant GHCH-10 MNPs were detached from the mould for further use.

### 2.4 Physiochemical characterizations

The morphology of the GHCH-n hydrogels and GHCH-10 MNPs was observed using a scanning electron microscope (SEM, SIGMA 500, Zeiss, USA). The chemical structures of GHCH-n hydrogels and raw materials were identified by Fourier transform infrared spectroscopy (FT-IR, Nicolet iS50, Thermo-Fisher, USA), X-ray diffraction (XRD, D8 advance, Bruker, Germany) and solid-state ^13^C NMR (Bruker Avance III 500 MHz). For FT-IR analysis, the wavenumbers used ranged from 4000 to 400 cm^−1^. For XRD analysis, the diffraction angles used ranged from 5° to 60°. For ^13^C NMR analysis, the chemical shift used ranged from 235 to −70 ppm. For the rheology test of the hydrogels, oscillation test and viscosity test were performed (Discovery DHR-2 (USA)) (oscillation-frequency scanning: 25 mm plate, 20 ℃ at constant 1% strain angular frequency: 1–100 s^−1^; flow-shear scanning: 25 mm plate, 20 ℃ at the shear rate: 1–100 s^−1^). The swelling kinetics of the GHCH-n hydrogels were detected following a previous report [41]. The compression tests were carried out using a universal material testing machine (HD-B609B-S, Haida, China). The stress‒strain curves and compressive strength at 35% strain were analysed.

### 2.5 Degradation test *in vitro*

The GHCH-n hydrogels were cut into pieces for degradation test *in vitro*. All the samples were freeze-dried and weighted (W1) before use. The samples were then incubated with 10 mL lysozyme solution (5 mg/mL) at 37 ℃. The lysozyme solution was replaced every day. Atregular time intervals, the samples were freeze-dried again and weighed (W2). The degradation rate (DR) of the hydrogels was calculated as follows:

(1) $$\text{DR}\;=\;\frac{W_{1}-W_{2}}{W_{1}}\times 100\%$$

### 2.6 Biocompatibility evaluation *in vitro*

#### 2.6.1 Haemolysis test

This study was performed with the approval of the Animal Care & Welfare Committee of Wuhan University. A healthy New Zealand rabbit was obtained from the Experimental Animal centre of Three Gorges University. After anaesthetization by isoflurane inhalation, the animal was fixed onto a corkboard to collect fresh anticoagulant blood, which was then diluted with 0.9% normal saline at a weight ratio of 1:1.25. The GHCH-n hydrogels were incubated with 10 mL normal saline and 200 µL diluted blood at 37 ℃ for 60 min. The positive and negative control groups were set up according to our previous report [42]. All samples were centrifuged at 1500 rpm for 10 min to separate the supernatants. The absorbence of the supernatants at 545 nm was detected by a multifunctional microplate reader (Spectrum M2, MD, USA).

#### 2.6.2 Preparation of GHCH-n extracts

The extracts of GHCH-n hydrogels were prepared under the guidance of ISO10993–12:2007. Briefly, the GHCH-n hydrogels were freeze-dried at −60 ℃ for 48 h and then completely sterilized by ultraviolet (UV) irradiation. The samples (0.2 g) were incubated with an FBS-containing medium at 37 ℃ for 72 h to obtain 1 mL extracts. The extracts were filtered using a 0.22 µm filter and stored at −80 ℃ for further study.

#### 2.6.3 MTT assay

In this study, three different cell lines (L929, BMSC, and HUVEC) were used as model organisms to evaluate the cytocompatibility of the GHCH-n hydrogels. The cells were seeded onto 96-well tissue culture plates at a density of 1.5–3.0 × 10^3^ cells/well. After cell adhesion overnight, the culture medium was replaced with a fresh medium containing 25% of the extracts. The blank control was treated with a culture medium containing no extracts. At regular time intervals, the samples were treated with MTT reagent according to the manufacturer's protocols. The absorbence at 490 nm was detected by a multifunctional microplate reader.

#### 2.6.4 Morphological observation

The cells were seeded onto 6-well tissue culture plates. After cell confluence reached 30%, the culture medium was replaced with a fresh medium containing 25% of extracts and cultured for another 48 h. All samples were rinsed with PBS 3 times and then treated with a live/dead cell staining kit. Live cells were stained with calcein-AM (green), and dead cells were stained with propidium iodide (red). An inverted fluorescence microscope (IX73, Olympus, Japan) was used for data acquisition.

### 2.7 Biocompatibility evaluation *in vivo*

Fifteen female Sprague‒Dawley (SD) rats weighing approximately 180 g were obtained from the Experimental Animal centre of Three Gorges University. Before evaluations *in vivo*, all rats were kept in the specific pathogen-free (SPF) Experimental Animal centre of Wuhan University for at least 7 days to relieve environmental stress. A distinctive intraperitoneal transplantation model was constructed for biocompatibility evaluations *in vivo* [43]. GHCH-n hydrogels (1 cm × 1 cm) were transplanted into the abdominal cavity of SD rats, followed by conventional feeding for 14 days. The animals were anaesthetized by isoflurane inhalation. Fresh whole blood was collected into pro-coagulation tubes and then centrifuged at 3000 rpm for 10 min to separate the plasma. A series of biochemical indices of plasma were automatically detected by the clinical laboratory. Meanwhile, the GHCH-n hydrogels as well as the organs (heart, liver, spleen, lung, and kidney) were resected and fixed in a 4% paraformaldehyde (PFA) solution. An H&E staining assay was performed to visualize the histopathological characteristics. An inverted fluorescence microscope was used for data acquisition. The project involves the use of experimental animals, which have been reviewed and approved by the Hubei University Animal Ethics and Welfare Committee.

### 2.8 Haemostasis tests

Thirty-six female New Zealand rabbits weighing approximately 2.5 kg were anaesthetized by isoflurane inhalation and then fixed on a surgical corkboard. Their abdominal cavity was opened using sterile surgical instruments to expose the organs, including the liver, spleen, and kidney. Herein, three different bleeding models were constructed for haemostasis evaluations of the GHCH-10 MNPs. For the liver and kidney bleeding model, the organs were punctured by a 50 mL syringe needle. For the spleen bleeding model, a surgical wound of 5 mm in length was made using ophthalmic scissors. Immediately after bleeding, the wound site was covered with a piece of GHCH-10 MNPs. In this study, a commercial gelatine sponge was used as a positive control, and medical gauze was used as a negative control. The blank control group was not treated. According to a previous report [44,45], bleeding time and blood loss were used for quantitative analysis. At least three independent samples were recorded.

### 2.9 Wound healing assay

Twelve female SD rats weighing approximately 180 g were safely anaesthetized by isoflurane inhalation. The hair on the back of the animals was removed, and for each animal, four round full-thickness skin defects of 15 mm in diameter were created. To construct the infected wound healing model, the proliferative *E. coli* bacterial suspension was directly applied to each wound. For the experimental group (GHCH-10), the wounds were covered with GHCH-0 or GHCH-10 MNPs and then securely fixed by surgical sutures. The negative control group was treated with medical gauze, and the positive control group was treated with commercial hydrocolloid dressings (Hyd.).

At regular time intervals, the optical images of wound sites were photographed using a digital camera (iPhone 13, China). Image-Pro Plus (IPP) 6.0 software was used to analyse the area of each wound. The wound healing rate (WHR) was calculated as follows:

(2) $$WHR\left(\%\right)=\frac{A_{0}-A_{t}}{A_{0}}\times 100$$

where A_0_ and A_t_ indicate the initial wound area and residual wound area at specific time points, respectively.

On Day 12, all animals were sacrificed. The neoskin tissue and organs (heart, liver, spleen, lung, and kidney) were resected and then fixed with 4% PFA solution for 72 h. For skin regeneration evaluations, H&E staining, Masson's staining, immunohistochemical staining (CD45, collagen I, and collagen III), and immunofluorescence staining (Ki67 and CD31) were performed according to standard protocols. For biocompatibility evaluations, H&E staining of the organs was also performed. A laser confocal microscope (LCS-SP8-STED, Leica, Germany) was used for high-resolution observation.

### 2.10 Statistical analysis

All data were quantitatively analysed using SPSS 23.0 software incorporated with a one-way analysis of variance. The results are shown as the mean ± standard deviation (SD). At least 3 independent samples were used for the statistical analysis. *P* < 0.05 indicated a significant difference.

## 3 Results and discussion

### 3.1 Fabrication and characterization of GHCH-n hydrogels

The ^13^C NMR spectrum of GelMA is presented in Fig. S1a. The chemical shifts of GelMA varied from 17.2 to 171.9 ppm, indicating there are many amino residues in gelatine. For example, the chemical shift at 41.1 ppm indicated the Arg-residue, which is one of the gelatine constitutions; the chemical shifts at 58.4 and 69.7 ppm indicated the Phe-residue, and the chemical shift at 171.9 ppm indicated the Lys, Leu-and Ala-residues. Besides, the chemical shifts of the C = C double bond are about 128 ppm and 135 ppm for both C = C atoms. Following a referenced gelatine amino acid constituent ratio, we calculated the ratio of C = C carbon atoms to all carbon atoms (R_1_) and the ratio of amino group attached carbon atoms that could be conjugated with methacrylate anhydride for the substitution (R_2_). The substitution degree (R) is presented as *R* = R_1_/R_2_ × 100%, and that is about 29.1%. As shown in Fig. S1b, for the ^13^C NMR spectrum of QCS, the chemical shifts of carbon atoms from glucosamine of chitosan backbone were presented at 61.1 ppm, 74.1 ppm, 83.1 ppm and 102.6 ppm respectively. Besides, the chemical shift of the methyl group from quaternary ammonium is about 54.3 ppm, and the value of spectral peak intensity integration is calculated as V_1_. The amino group attached to the carbon atom from glucosamine from the chitosan backbone is about 83.1 ppm, which could react with glycidyl trimethylammonium chloride for the substitution, the value of spectral peak intensity integration is calculated as V_2_. Thus, the substitution degree of QCS is calculated as *R* = V_1_/V_2_ × 100%, and that is about 91.3%.

GelMA/QCS composite hydrogels (GHCH-n) were created using UV-mediated crosslinking with the help of a photo-initiator (L2959). GelMA served as a stiff molecular skeleton. QCS was immobilized into the GelMA network by the synthetic molecular cage effect and using intermolecular hydrogen. As a result, GHCH-n can be categorized as a conventional semi-interpenetrating hydrogel (S-IPH). QCS was positively charged and imparted desirable bioactive activity to the GHCH-n hydrogels.

**Fig. 2:**
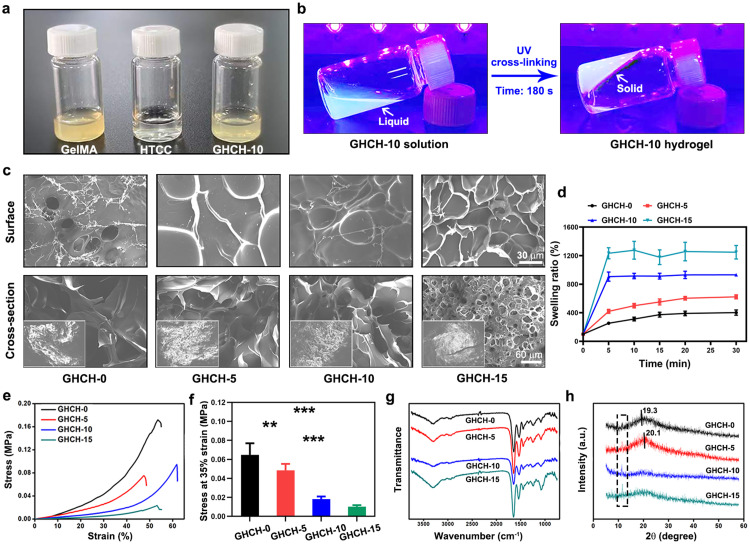
**Structure and physiochemical properties of GHCH-n hydrogels.** (a) Optical images of the GHCH-10 solution and raw materials; (b) After UV cross-linking at a power of 500 W for 180 s, the GHCH-10 hydrogel was successfully prepared; (c) Superficial and cross-sectional morphology of the GHCH-n hydrogels observed by SEM, scale bar: 30 or 60 µm; (d) Swelling kinetics of the dried GHCH-n hydrogels; (e) Stress‒strain curves of the GHCH-n hydrogels; (f) Quantitative results of compressive strength at 35% strain; (g) FT-IR spectrum of the GHCH-n hydrogels; (h) XRD spectrum. Values are expressed as the mean ± SD (*n* = 3), **P < 0.01, ***P < 0.001.

The fabrication processes of GHCH-n hydrogels are shown in Fig. 2a,b. Briefly, the GelMA solution and QCS solution were blended at a specific proportion. The as-gelled solution was then exposed to UV light at a power of 500 W for 180 s. A liquid-to-solid phase transition occurred. In the meantime, a minor quantity of solvent precipitated, indicating the creation of a cross-linked biomolecular network.

The GHCH-n hydrogels were freeze-dried and then observed by SEM. As shown in Fig. 2c, the structure of the GHCH-n hydrogels was compact at the surface but porous on the interior. With an increase in QCS content (*n* = 0, 5, 10, 15), the surface morphology of GHCH-n becomes rough, and the internal pore size decreases gradually. This phenomenon suggested that the incorporation of QCS increased the degree of chemical crosslinking in GHCH-n hydrogels and enhanced the intermolecular force [46].

The swelling ability of GHCH-n hydrogels is shown in Fig. 2d. All of the GHCH-n groups absorbed a large amount of water in the first 5 min and quickly entered the platform period. The equilibrium swelling ratio at 30 min was 401.8% ± 36.8% for the GHCH-0 group, 622.8% ± 27.7% for the GHCH-5 group, 931.3% ± 10.7% for the GHCH-10 group, and 1247.9% ± 95.2% for the GHCH-15 group. Significant differences between each group were observed (*P* < 0.05). In this study, the quantity of QCS had a significant impact on the swelling ability [47]. The QCS component of the hydrogels was more hydrophilic than the GelMA component; thus, it may play a dominant role in the swelling ability of the hydrogels.

For the oscillation test, the values of storage modulus (G’) of all samples were greater than that of loss modulus (G”) (Fig. S2), which indicated that these samples were in elastic states. These results also confirmed the formation of UV crosslinked hydrogels. A compressive test was also performed to evaluate the mechanical properties of the GHCH-n hydrogels. As shown in Fig. 2e, the compressive stress increased gradually with increasing strain in each group. To quantitatively reflect the mechanical strength, the compressive stress at 35% strain was statistically analysed. As shown in Fig. 2f, the compressive stress was 0.065 ± 0.012 MPa for the GHCH-0 group, 0.048 ± 0.007 MPa for the GHCH-5 group, 0.018 ± 0.003 MPa for the GHCH-10 group, and 0.010 ± 0.002 MPa for the GHCH-15 group. Significant differences between each group were observed (*P* < 0.05). The mechanical strength of hydrogels is highly correlated with the rigidity of biomolecules as well as the degree of chemical cross-linking [48]. Herein, QCS did not participate in the chemical crosslinking reaction, and the incorporation of QCS directly led to the weakness of the GHCH-n hydrogels. This phenomenon could be largely attributed to the decreased intensity of chemical cross-linking amongst the network molecular structures of GelMA and QCS.

The chemical compositions of the GHCH-n hydrogels were characterized by FT-IR and XRD analysis. As shown the FT-IR results in Fig. 2g, The peaks of 1531, 1446, 1638, and 3293 cm^−1^ were observed in the spectrum of GHCH-0. The peaks at wavenumber around 1638 cm^−1^, 1531 cm^−1^ and 1446 cm^−1^ were attributed to amide I, amide II and amide III respectively, which were corresponding to the stretching of C = O bond, bending of N—H bond and plane vibration of C—N and N—H. The broad peak at 3293 cm^−1^ was related to the stretching of the hydrogen-bonded hydroxyl groups [49,50]. These results confirmed the existence of GelMA. For the QCS component, the weak band at 2929 cm^−1^ was related to the –CH– stretch of chitosan, and the –NH and –OH stretching vibrations as well as the inter- and extramolecular hydrogen bonds of chitosan molecules were observed at approximately 3320 cm^−1^ to 3500 cm^−1^ [51]. For GHCH-5, GHCH-10, and GHCH-15, the characteristic peaks of GHCH-0 described above were also present, and the intensities of these peaks progressively increased with increasing QCS content. As shown in Fig. 2h, the XRD spectrum of all GHCH-n hydrogels indicated the disappearance of peaks at approximately 19.3° to 20.1° as the QCS content increased. This phenomenon can be explained by the positively charged QCS segment disrupting the intermolecular hydrogen bonding between GelMA molecules. A sharp peak increased gradually from GHCH-0 to GHCH-15 (dotted box), indicating that an unknown crystalline GelMA was strengthened as the QCS content increased. This result confirmed the existence of QCS and the interaction between QCS and GelMA from one aspect.

### 3.2 GHCH-n hydrogels were biocompatible *in vitro*

**Fig. 3:**
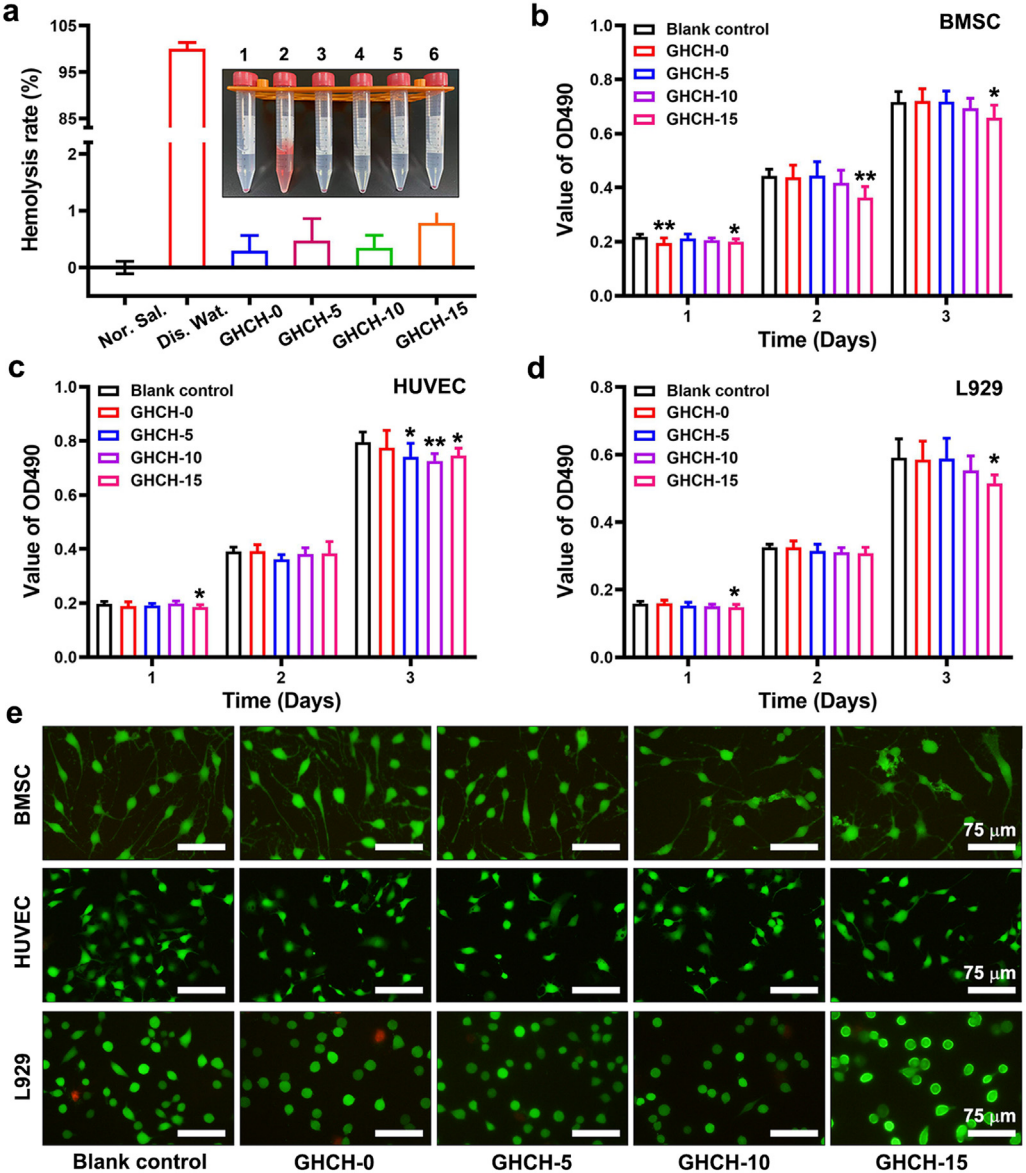
**GHCH-n hydrogels was haemocompatible and cytocompatible *in vitro*.** (a) Quantitative results of the haemolysis test; (b-d) The cell viability of BMSCs, HUVECs, and L929 cells after coincubation with the GHCH-n extracts for 3 days; (e) The morphology of extract-treated cells was visualized using a live/dead cell staining assay. Live cells were stained green, and dead cells were stained red. Scale bar: 75 µm. Values are expressed as the mean ± SD (*n* = 6). Compared to the blank control, *P < 0.05, **P < 0.01.

Next, the biocompatibility of GHCH-n hydrogels was evaluated from different dimensions. First, the GHCH-n hydrogels were coincubated and placed in direct contact with red blood cells at 37 ℃ for 60 min. Then, the haemolysis rate of red blood cells was quantitatively detected using light absorption methods. As shown in Fig. 3a, the haemolysis rate of the negative control (Nor. Sal.) was set as 0%, and that of the positive control (Dis. War.) was set as 100%. The haemolysis rate was 0.30% ± 0.27% for the GHCH-0 group, 0.48% ± 0.38% for the GHCH-5 group, 0.35% ± 0.22% for the GHCH-10 group, and 0.79% ± 0.33% for the GHCH-15 group. Thus, the haemocompatibility of the GHCH-n hydrogels could meet the high requirement of implantable medical devices (less than 5%) [52].

Extracts of GHCH-n hydrogels were prepared to evaluate cytocompatibility. The extracts contained a small number of soluble components, such as QCS and uncross-linked GelMA. Currently, the effect of the extracts on the proliferation and survival of normal cells remains to be studied. Herein, three different cell lines served as model organisms and were cultured with extract-containing medium for 24–72 h. The cell proliferation ability of the treated cells was evaluated by MTT assay. As shown in Fig. 3b-d, all cell types grew well in each group. As the value of n increased from 0 to 15, the value of OD490 decreased slightly. The mild cytotoxicity of GHCH-10 hydrogels could be attributed to the positively charged and amphiphilic QCS. QCS could be inserted into the targeted cell membrane to cause cell rupture and death [53,54]. Thus, the relative content of QCS in the GHCH-n hydrogels should be limited. Fortunately, the relative cell viability of the GHCH-n groups was significantly higher than 80% compared to the blank control (*P* < 0.05) [55]. A live/dead cell staining assay was performed as a supplement to the MTT assay. The cells were cultured with extract-containing medium for 48 h. Live cells were dyed green, and dead cells were dyed red. As shown in Fig. 3e, almost all cells were green and exhibited normal cell morphology. Based on the above results, it could be concluded that the GHCH-n hydrogels had relatively good cytocompatibility following the general standards.

### 3.3 GHCH-n hydrogels were biocompatible *in vivo*

**Fig. 4:**
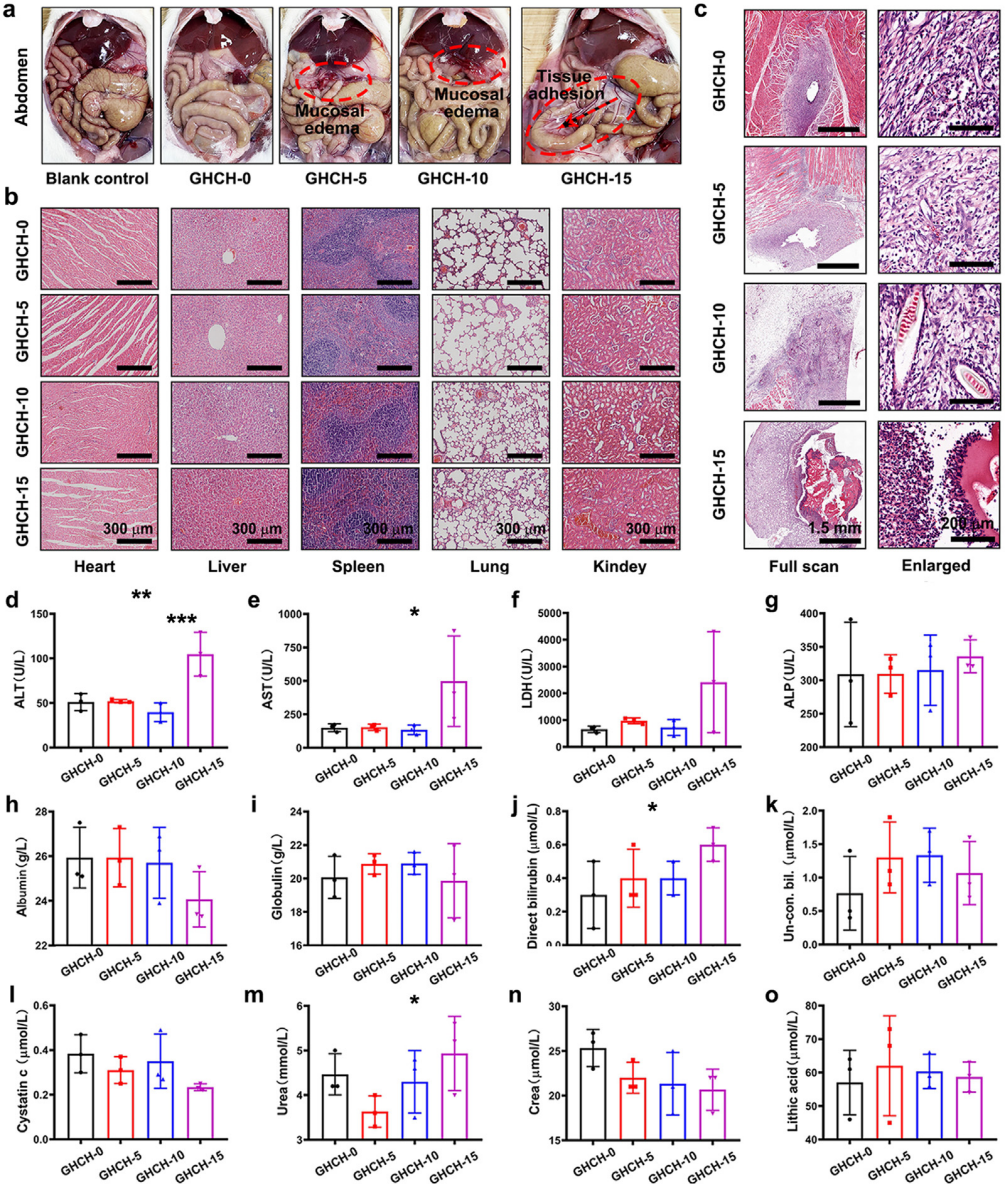
**GHCH-10 exhibited the best histocompatibility *in vivo*.** (a) GHCH-n hydrogels were transplanted into the abdomen of SD rats, leading to mild immune rejection; (b) The organs of animals were not damaged by transplantation of GHCH-n hydrogels. Scale bar: 300 µm; (c) In all but the GHCH-15 experimental group, the GHCH-n hydrogels and surrounding fibrous capsules showed less tissue inflammation than the tissue at the wound site in the control group. Scale bar: 1.5 mm or 200 µm; (d-o) A series of blood biochemical indices were tested. Values are expressed as the mean ± SD (*n* = 3), *P < 0.05, **P < 0.01, ***P < 0.001.

The biocompatibility of GHCH-n hydrogels *in vivo* was further evaluated using a distinctive intraperitoneal transplantation model [43]. GHCH-n hydrogels were implanted into the abdominal cavity of SD rats for 14 days, as illustrated in Fig. 4a. To defend against foreign body rejection, the larger omentum migrated and enveloped the hydrogels. GHCH-5 and GHCH-10 showed minor mucosal oedema when compared to the blank control. The GHCH-15 group, on the other hand, experienced substantial tissue adhesion. This incident further demonstrated that QCS is potentially hazardous.

To reveal the systematic side effects, the organs of treated SD rats were resected for histological evaluations. The optical images of H&E staining are shown in Fig. 4b. The GHCH-0, GHCH-5, and GHCH-10 groups exhibited normal histological characterizations. However, the liver of the GHCH-15 group showed typical central venous coagulation. Meanwhile, the kidneys of the GHCH-15 group showed diffuse glomerular atrophy and necrosis. Thus, the hepatorenal toxicity of GHCH-15 hydrogels should be given more attention.

The GHCH-n hydrogels and the capsule tissue were further characterized by H&E staining. As shown in Fig. 4c, the GHCH-15 group exhibited massive inflammatory cell infiltration and local tissue necrosis. This result was consistent with those presented in Fig. 4a,b. A series of biochemical indices were also detected, as shown in Fig. 4d-o and Fig. S4. Most indices were within the normal range. However, the alanine aminotransferase (ALT), aspartate aminotransferase (AST), and lactate dehydrogenase (LDH) levels of the GHCH-15 group were significantly higher than those of the other groups (*P* < 0.05). These results also confirmed the liver injury induced by QCS.

Based on the above results, we found that QCS was potentially toxic to the liver and kidney. The side effects of the GHCH-15 hydrogel were too serious to be used clinically, but those of the GHCH-5 and GHCH-10 hydrogels were still within the controllable and acceptable range. This phenomenon might be attributed to the dose-dependent effect of QCS. Herein, a degradation test *in vitro* was performed to verify this hypothesis. As shown in Fig. S5, the degradation rate increased gradually from GHCH-0 to GHCH-15. The fast degradation is conducive to the release of QCS. QCS is like a two-edged sword, but the releasing dynamics could be easily adjusted by controlling the content of QCS. To optimize the composition to find the best candidate, we performed a broad-spectrum antibacterial test. As shown in Fig. S6, the GHCH-n (*n* = 5, 10, 15) hydrogels showed relatively good antibacterial activity toward both *E. coli* and *S. aureus*. As the value of n increased from 5 to 15, the antibacterial activity of the GHCH-n hydrogels was greatly enhanced. Similar results have been reported in a large number of studies [41,56]. Thus, the GHCH-10 hydrogel was selected for further translational research.

### 3.4 GHCH-10 MNPs were haemostatic *in vivo*

In recent decades, microneedle patches (MNPs) have been of increasing interest, especially in the field of wound healing [57]. The microneedle array of MNPs can not only deliver therapeutic drugs through the skin but also firmly fix the MNPs onto wound sites [58]. The excellent tissue adhesion of MNPs makes them an ideal biomaterial for non-compressive haemostasis. QCS is positively charged and can shield the negative charge on the surface of red blood cells [59]. Thus, the incorporation of QCS could endow the GHCH-10 hydrogel with potential haemostatic activity. It is assumed that the haemostatic effect and clinical competitiveness of the GHCH-10 hydrogel would be greatly improved if it is processed into MNPs.

**Fig. 5:**
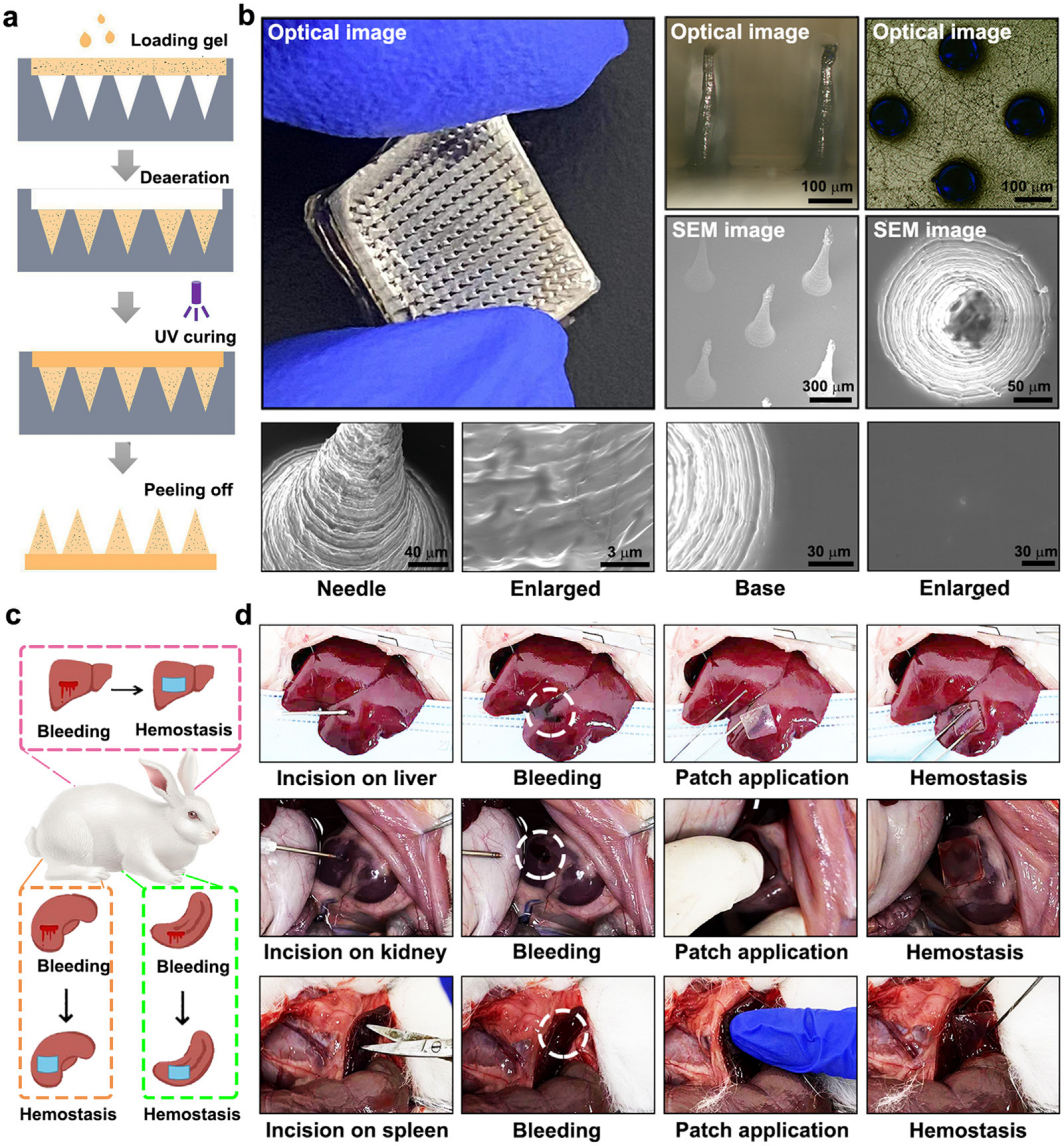
**GHCH-10 was shaped as microneedle patches (MNPs) for non-compressive haemostasis.** (a) The preparation method of GHCH-10 MNPs; (b) The microstructure of GHCH-10 MNPs was observed by metallurgical microscopy and SEM. Scale bars are marked. (c) Three different bleeding models were constructed for haemostasis evaluations *in vivo*. (d) The haemostasis effect of GHCH-10 MNPs was recorded.

As shown in Fig. 5a, GHCH-10 MNPs were prepared using a mould-forming method. The global and local morphologies of GHCH-10 MNPs are shown in Fig. 5b. The GHCH-10 MNPs could be divided into needles and bases. The surface of the needle showed a concentric circle texture, and that of the base was relatively smooth. This phenomenon could be attributed to the phase separation of hydrogels during the drying process. As shown in Fig. S7, the GHCH-10 MNPs were stiff enough to puncture skin tissue.

As shown in Fig. 5c, three different models, including the liver bleeding model, spleen bleeding model, and kidney bleeding model, were constructed to evaluate the haemostatic effect of GHCH-10 MNPs. Immediately after bleeding, a piece of GHCH-10 MNPs was placed onto the wound site, followed by momentary pressing. As shown in Fig. 5d, the GHCH-10 MNPs adhered to the wounds and quickly sealed the bleeding site. In this study, the GHCH-10 MNPs also electroactively interacted with the blood components to accelerate the formation of blood clots. As shown in Table 2, the haemostatic effect of GHCH-10 MNPs was also quantitatively analysed by bleeding time and blood loss. Notably, the haemostatic effect of GHCH-10 MNPs was comparable to that of commercial haemostatic materials (gelatine sponges).

**Table 2:** **Haemostatic effect of GHCH-10 MNPs in three bleeding models (mean ± SD,*****n*****=3)**.

	Liver bleeding model		Spleen bleeding model		Kidney bleeding model	
	Bleeding time (s)	Blood loss (g)	Bleeding time (s)	Blood loss (g)	Bleeding time (s)	Blood loss (g)
Blank control	205.3 ± 22.7	7.2 ± 1.2	79.3 ± 5.5	0.82±0.10	177.7 ± 17.6	4.17±0.64
Medical gauze	86.3 ± 4.5^$^	3.1 ± 0.9^$^	55.7 ± 5.7^$^	0.43±0.07^$^	137±8.7^$^	3.07±0.35^$^
Gelatine sponges	75.7 ± 11.1^$^	1.9 ± 0.3^$^	36.3 ± 3.5^$, #^	0.36±0.05^$^	124.3 ± 7.5^$^	2.13±0.21^$, #^
GHCH-10 MNPs	73.7 ± 6.7^$, #^	1.7 ± 0.6^$^	31.3 ± 2.5^$, #^	0.34±0.07^$^	108.7 ± 7.4^$, #^	2.17±0.38^$, #^

Compared to blank *control, $P* < 0.05; compared to medical gauze, ^#^*P* < 0.05.

### 3.5 GHCH-10 MNPs accelerated infected wound healing

**Fig. 6:**
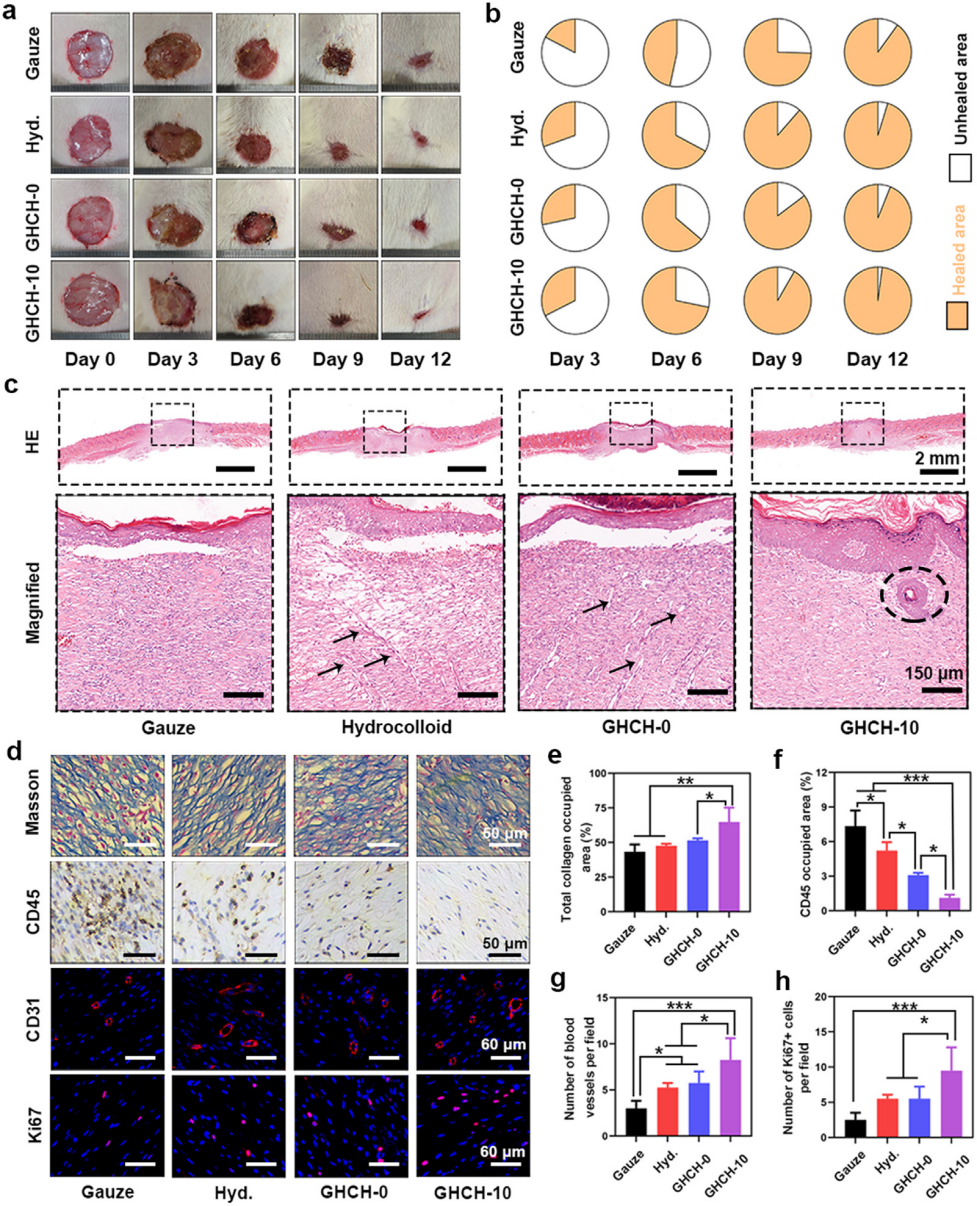
**GHCH-10 MNPs were further applied to full-thickness wound healing.** (a) Images of wound sites at different time points; (b) Fractions of the healed area; (c) H&E staining images of the regenerated skin tissue at Day 12. The arrow indicates immature sinuses, and the circle indicates hair follicles. Scale bar: 2 mm or 150 µm; (d) Immunohistochemical (IHC) and immunofluorescent (IF) images of the regenerated skin tissue at Day 12. Scale bar: 50 µm or 60 µm; (e-h) The quantitative results of histological analysis. Values are expressed as the mean ± SD (*n* = 3), *P < 0.05, **P < 0.01, ***P < 0.001.

An *E. coli*-infected full-thickness skin injury model was successfully constructed to identify the promotion effect of GHCH-10 MNPs on wound healing. The initial wound size was set to 15 mm in diameter, and the wound healing period lasted for almost 12 days. Herein, the negative control was treated with biologically inert medical gauze, and the positive control was treated with a bioactive and antibacterial Ag^+^-containing hydrocolloid dressing. At regular time intervals, the wound sites were photographed to calculate the wound size [60]. As shown in Fig. 6a,b, the wound sites healed gradually in each group within 12 days. The GHCH-10 group healed faster than the other groups at each time point. For infected wound healing, rapid contraction of the wound is essential to prevent secondary infection and injury [1,61].

On Day 12, the neoskin tissue was collected for standardized histological characterizations. The results of H&E staining are shown in Fig. 6c. Immature granulation tissue was marked and magnified. The Gauze group was infiltrated with mostly inflammatory cells, partly owing to the poor biocompatibility of cotton fibres. A large number of immature sinuses were observed in the hydrocolloid group and GHCH-0 group, suggesting the proliferation phase of wound healing [62,63]. In the GHCH-10 group, a complete keratinized epithelium formed. The keratinized epithelium protrudes into the dermis, which is conducive to tissue connection and nutrient transportation [64]. However, the extracellular matrix (ECM) is synthesized in large quantities and remodelled. Skin accessories first appeared, such as hair follicles. In conclusion, the quality of wound healing in the GHCH-10 group was significantly better than that in the other groups in terms of histology.

A series of IHC and IF staining assays were performed to investigate the bioactivity of GHCH-10 MNPs. As shown in Fig. 6d-h, GHCH-10 MNPs promoted the deposition of collagen protein (Masson) and inhibited bacterial-induced inflammation (CD45). It was confirmed that GHCH-10 MNPs have a significantly greater promotion effect on Col-I than on Col-III (Fig. S8). GHCH-10 MNPs also promoted host cell proliferation (Ki67) and vascularization (CD31). For each index, a significant difference was observed between the GHCH-10 group and the other groups (*P* < 0.05). After the wound-healing process, no obvious organ damage was observed in any of the treated animals, suggesting good biosafety of GHCH-10 MNPs (Fig. S9).

The effectiveness and safety of GHCH-10 MNPs have been validated in this section. The GHCH-10 MNPs have the potential to significantly speed wound contraction and enhance regeneration. GHCH-10 MNPs were found to be multifunctional and capable of interacting with the host in the time-dependent phase of wound healing. The application impact and translational potential of GHCH-10 MNPs are superior to those of medical gauze and hydrocolloid dressing. The GHCH-10 MNPs are expected to be expanded into a broader range of biomedical applications, including but not limited to wound dressings and haemostatic biomaterials.

## 4 Conclusion

To summarize, we initially used a UV cross-linking strategy to create a variety of GelMA/QCS composite hydrogels. The products' physiochemical characteristics, biocompatibility, and bioactivities (codes as GHCH-n, *n* = 0, 5, 10, and 15) were studied *in vitro* and *in vivo*. The GHCH-10 hydrogel outperformed the other three hydrogels and was used to fabricate microneedle patches (MNPs). The obtained GHCH-10 MNPs effectively reduced bleeding time and blood loss. GHCH-10 MNPs had haemostatic properties comparable to those of commercial gelatine sponges. Furthermore, GHCH-10 may hasten wound healing in *E. coli*-infected wounds by boosting collagen deposition, cell proliferation, and vascularization and suppressing bacterial inflammation. This work is of great significance for optimizing effective and safe biomaterials through the collaborative application of multidimensional research models. The product of this work will provide a potential substitute for complicated wound healing.

## Declaration of competing interest

The authors declare that they have no conflicts of interest in this work.

## Appendix Supplementary materials

Image, application 1
