# Supplementary material for: Facile fabrication of quaternized chitosan-incorporated biomolecular patches for non-compressive haemostasis and wound healing
Source: Fundam Res. 2023 May 30;4(5):1243–53. doi: 10.1016/j.fmre.2023.05.009 (PMC11489470; doi:10.1016/j.fmre.2023.05.009)
Supplement: Supplementary file 1 [file mmc1.docx]

**Supplementary information**

**Facile fabrication of quaternized chitosan-incorporated biomolecular patches for non-compressive haemostasis and wound healing**

Zesheng Chen^a,b,1^, Yixuan Zhang^c,1^, Kexin Feng^b^, Tao Hu^b^, Bohan Huang^b^, Jinlan Tang^b^, Junjie Ai^d^, Liang Guo^c,*^, Weikang Hu^b,*^ and Zijian Wang^a,b,^[[1]](#footnote-1)^*^

^a^ Department of Urology, Cancer Precision Diagnosis and Treatment and Translational Medicine Hubei Engineering Research Center, Zhongnan Hospital of Wuhan University, Wuhan 430071, China

^b^ Ministry of Education Key Laboratory of the Green Preparation and Application for Functional Materials, Hubei Key Laboratory of Polymer Materials, School of Materials Science and Engineering, Hubei University, Wuhan 430062, China

^c^ Department of Plastic Surgery, Zhongnan Hospital of Wuhan University, Wuhan 430071, China

^d^ Department of Clinical Laboratory, Hubei Provincial Hospital of Traditional Chinese Medicine, Wuhan 430061, China

**Experimental methods**

**Chemical synthesis of QCS**

Quaternized chitosan (QCS) was synthesized according to previous report with some modification[1]. Chitosan powder (2.0 g) was dissolved into 98 g of 0.5% AcOH solution with constant stirring. GTMAC (15 g) was then added into the mixed solution, and reacted at 55 ℃ for 18 h. The products were precipitated and purified by acetone for three time, and then freeze-dried for further study.

**Chemical synthesis of GelMA**

Mathacylated gelatin (GelMA) was synthesized according to previous report[2]. Gelatin solution was prepared by adding 10 g of gelatin into 90 g of deionized water. After stirring at 50 ℃ for 30 min, gelatin was completely dissolved. MA solution (10 g) was dropped into the gelatin solution, and then reacted for another 3 h. The products were dialyzed for 3 days to remove the unbound small molecules, and then freeze-dried for further study.

**Antibacterial assay**

In this study, gram-negative bacteria *Escherichia coli* (*E. coli*) and gram-positive bacteria *Staphylococcus aureus* (*S. aureus*) were both used as model organism of antibacterial assay. The GHCH-n hydrogels with 1 × 1 cm^2^ in size were co-incubated with 10 mL bacterial suspensions with constant stirring. At regular time interval, the absorbance of bacterial suspension at 600 nm was detected using a nanodrop ultraviolet spectrophotometer (ND-ONE-W, Thermo-Fisher, USA).


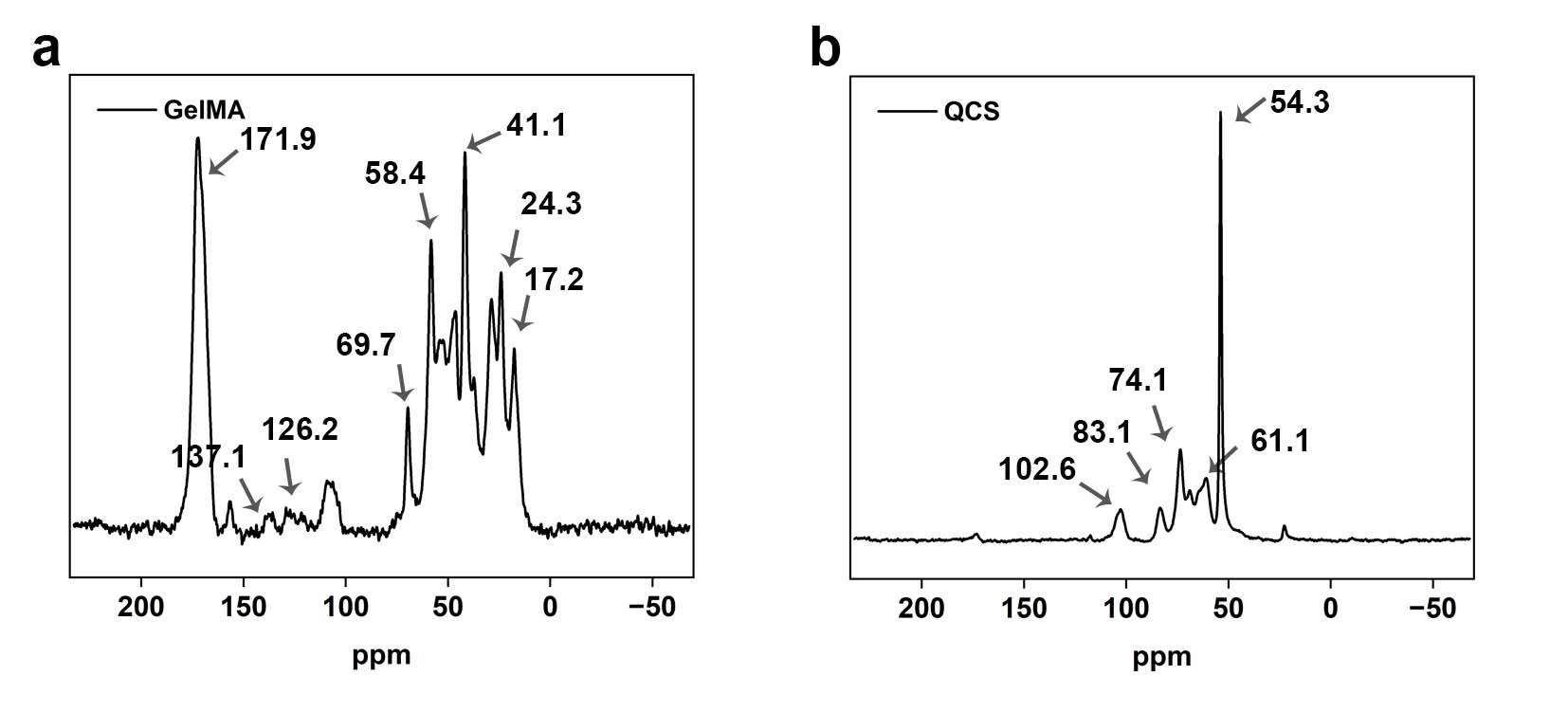


**Fig. S1.** (a) The ^13^C-NMR spectrum of GelMA; (b) The ^13^C-NMR spectrum of QCS


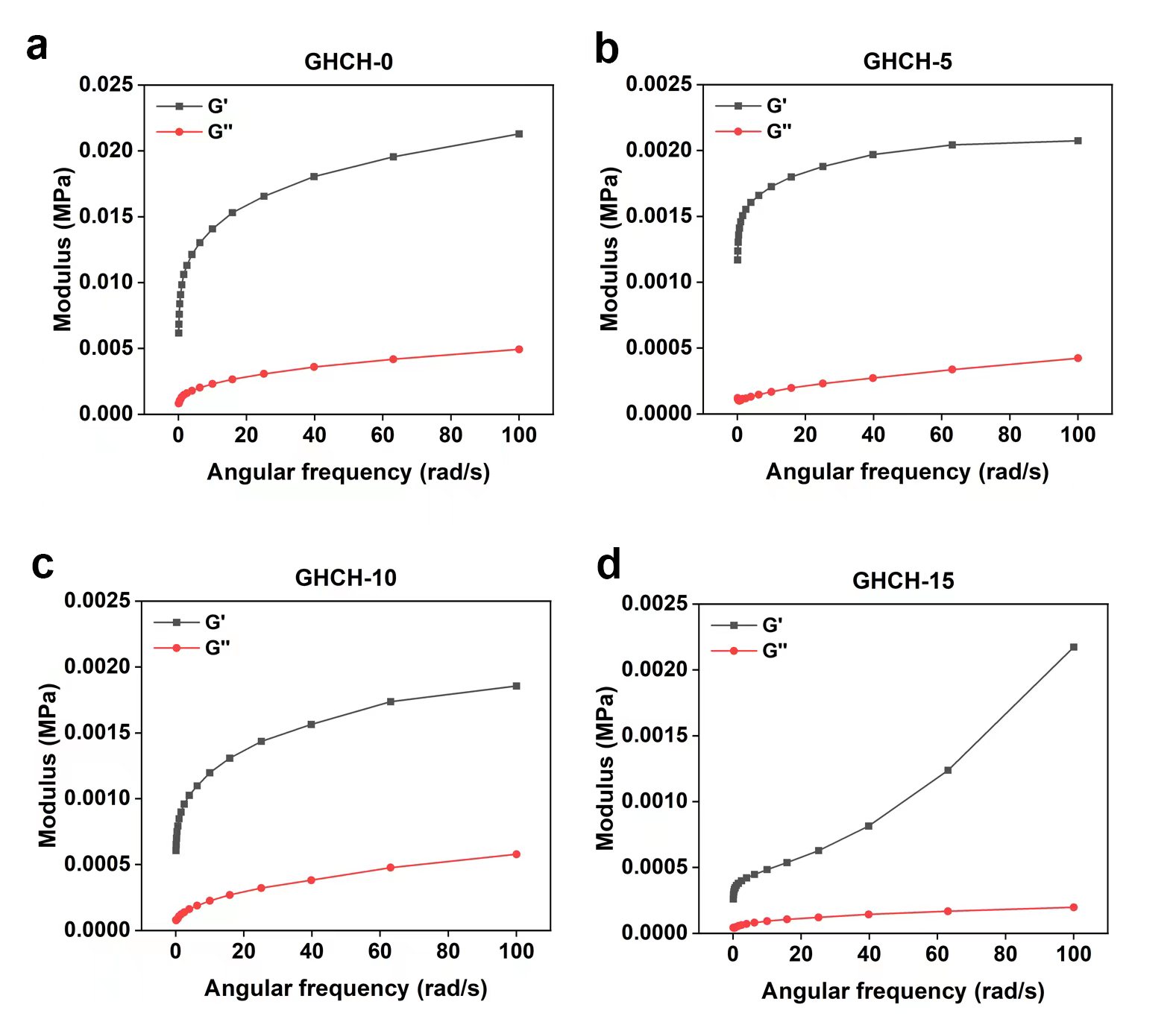


**Fig. S2.** The oscillation test of all the GHCH samples





**Fig. S3.** The viscosity tests of all the GHCH samples


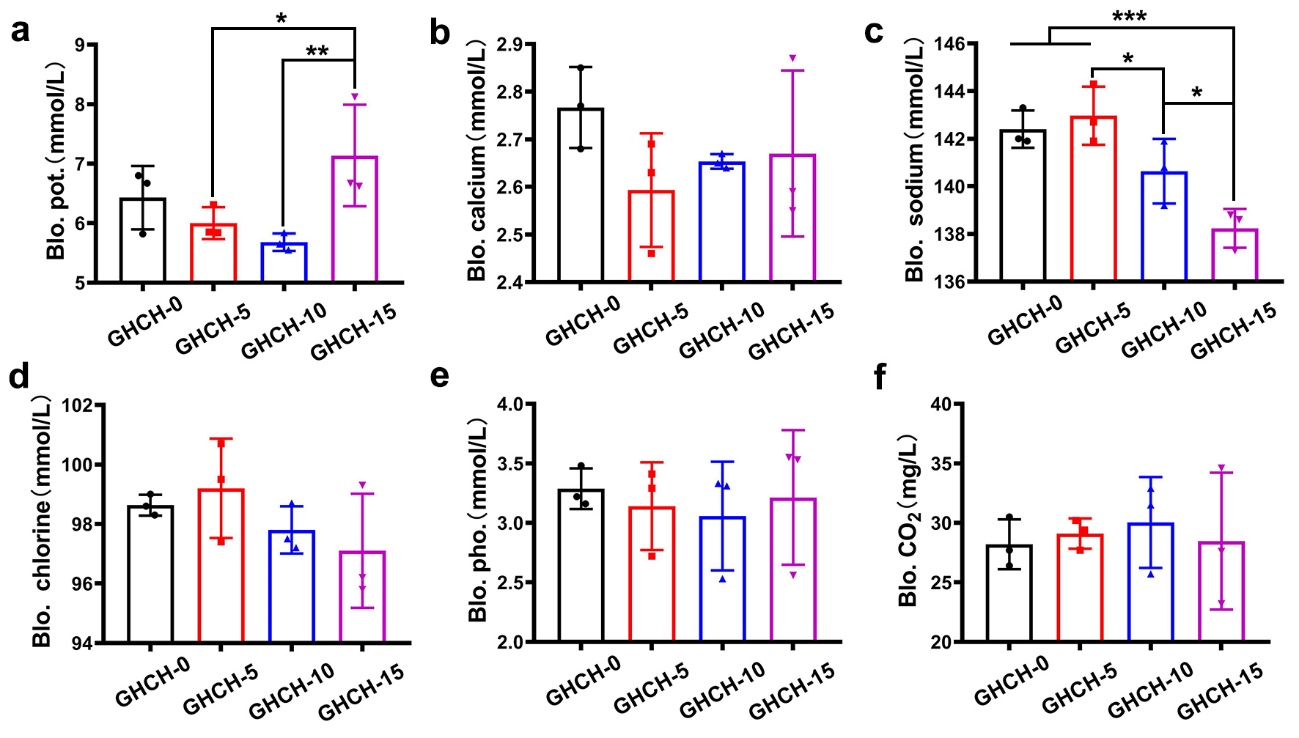


**Fig. S4.** (a-f) A series of blood biochemical indexes were tested. Values are expressed as the mean ± SD (*n* = 3), **P* < 0.05, ***P* < 0.01, ****P* < 0.001.





**Fig. S5.** The vitro degradation tests of all the GHCH samples


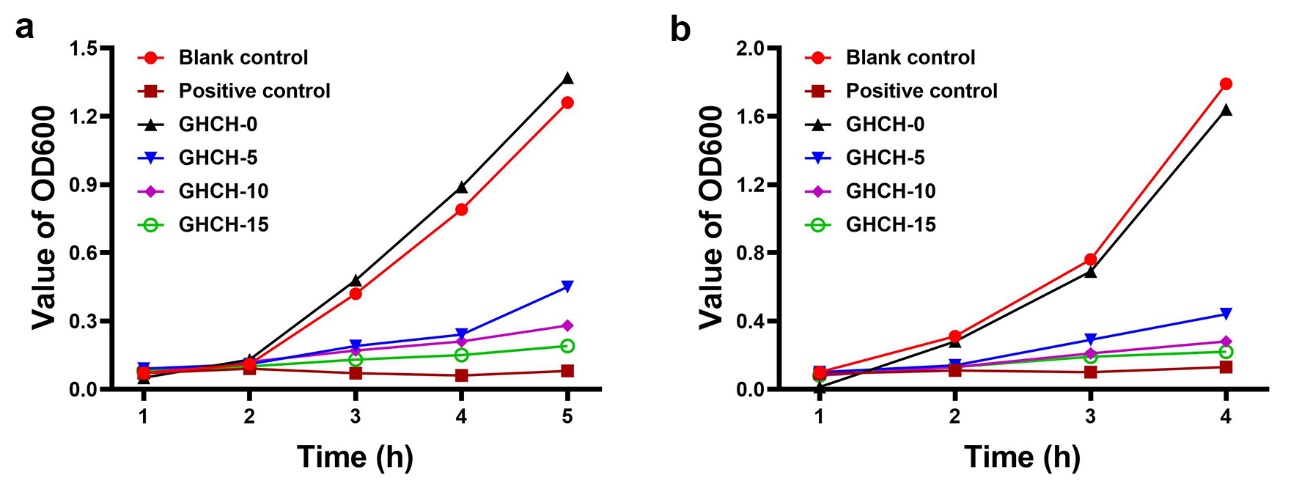


**Fig. S6.** (a) The proliferation curves of *E. coli* bacteria after treated by GHCH-n; (b) The proliferation curves of *S. aureus* bacteria after treated by GHCH-n. Blank control was treated with 0.85% normal saline, and positive control was treated with ampicillin solution.


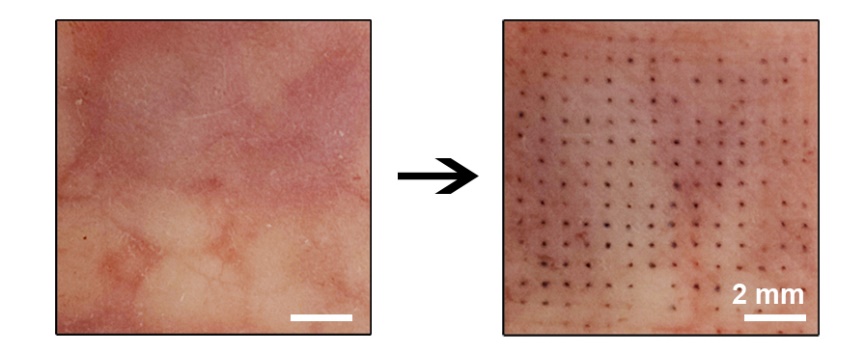


**Fig. S7.** GHCH-10 MNPs punctured the skin of mouse.


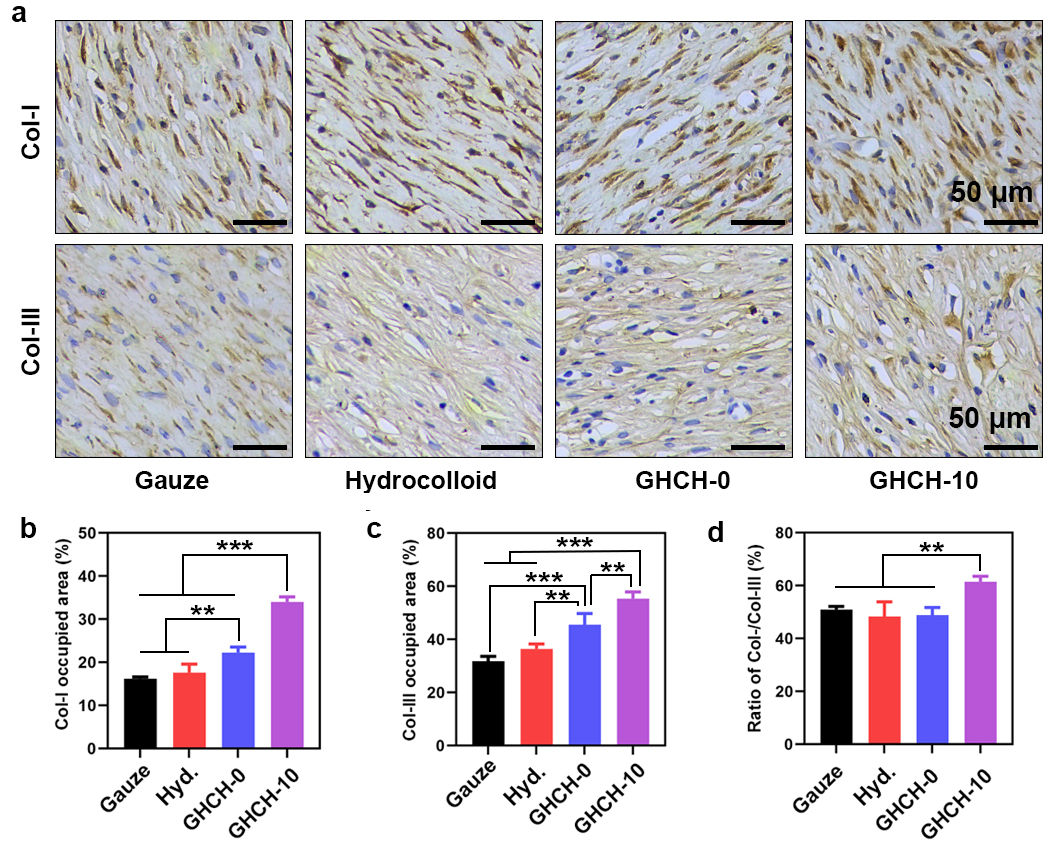


**Fig. S8.** (a) Immunohistochemical (IHC) staining images of the regenerated skin tissue at day 12. Scale bar: 50 μm; (b-d) The quantitative results of histological analysis. Values are expressed as the mean ± SD (*n* = 3), ***P* < 0.01, ****P* < 0.001.


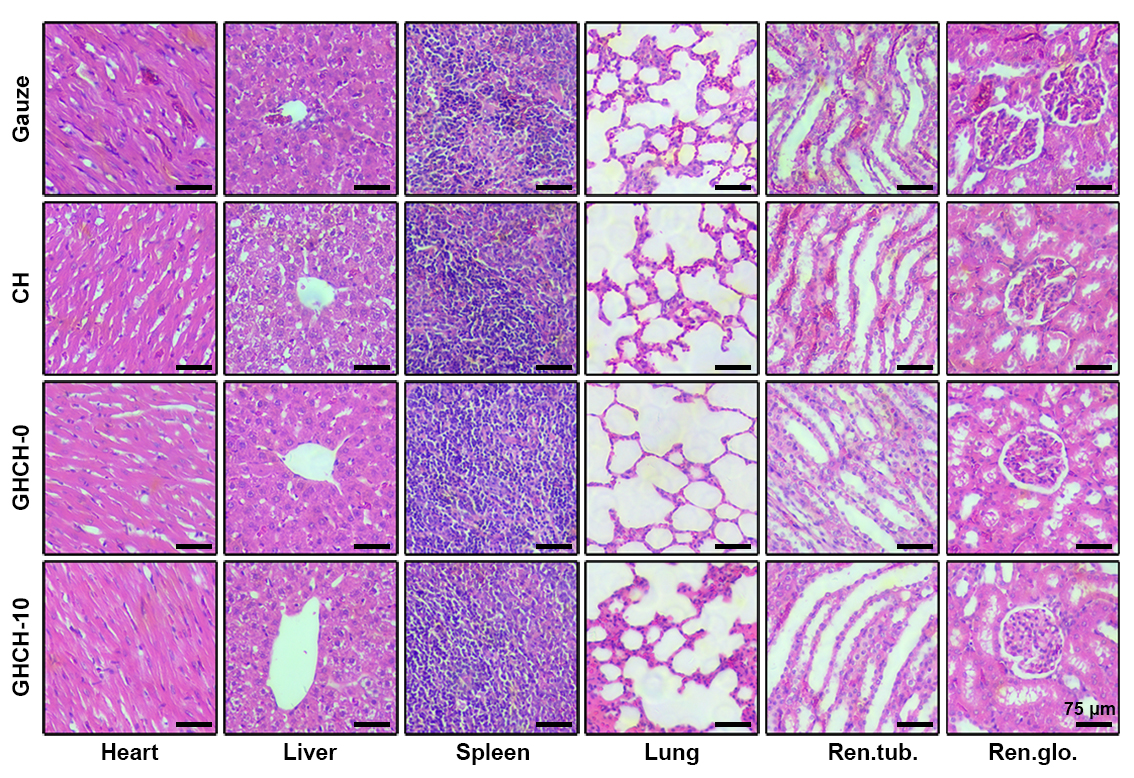


**Fig. S9.** HE staining images of organs. Scale bar: 75 μm. In this study, the animals were sacrificed after the wound healed, and the organs were collected for histological analysis.


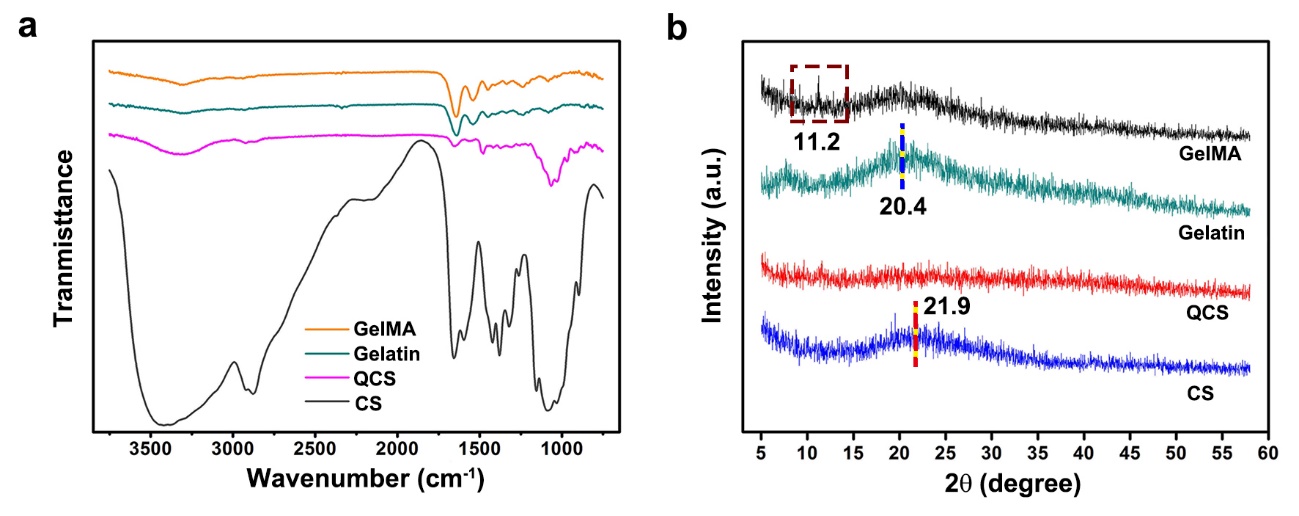


**Fig. S10.** (a) FT-IR spectrum of the raw materials, including mathacylated gelatin (GelMA), quaternized chitosan (QCS/HTCC), gelatin and chitosan (CS); (b) XRD spectrum of the raw materials.

**Reference**

[1] Huang RH, Yang BC, Zheng DS, Wang B. Preparation and characterization of a quaternized chitosan. *Journal of Materials Science*. **2012**, *47*, 845.

[2] Yue K, Trujillo-de Santiago G, Alvarez MM, Tamayol A, Annabi N, Khademhosseini A. Synthesis, properties, and biomedical applications of gelatin methacryloyl (GelMA) hydrogels. *Biomaterials*. **2015**, *73*, 254.

1. *Corresponding authors:

   E-mail address: [Zijianwang@whu.edu.cn](mailto:Zijianwang@whu.edu.cn) (Z. Wang); [Huwkang@hubu.edu.cn](mailto:Huwkang@hubu.edu.cn) (W. Hu); [Guoliang@whu.edu.cn](mailto:Guoliang@whu.edu.cn) (L. Guo).

   ^1^ These authors contributed equally to this work. [↑](#footnote-ref-1)
